# Primary Non-Hodgkin’s Lymphoma of Penis Masquerading as a Non-Healing Ulcer in the Penile Shaft

**DOI:** 10.5812/numonthly.6885

**Published:** 2013-05-28

**Authors:** Kushal Karki, Rehan Mohsin, Muhammed Mubarak, Altaf Hashmi

**Affiliations:** 1 Urology Department, Sindh Institute of Urology and Transplantation, Karachi, Pakistan; 2 Hisopatology Department, Sindh Institute of Urology and Transplantation, Karachi, Pakistan

**Keywords:** Biopsy, Lymphoma, Ulcer, Penis

## Abstract

Primary malignant lymphoma of the male external genitalia is extremely rare and it is even rarer in the penis. Because of its rarity, the possibility of delay in diagnosis and mismanagement is always there. It can present as a nodule, non-healing ulcer, stricture urethra or periurethral abscess. We report a case presenting first a nodule and later on as a non-healing ulcer which was diagnosed by corporal biopsy and managed successfully with chemotherapy.

## 1. Introduction

Primary malignant lymphoma of the male external genitalia is a rare disease (1, 2). It is most commonly reported in old age but one pediatric case has been reported in the literature (3). Herein we present a case of a 49-year-old man presenting with a lump at the base of the penis followed by a non-healing ulcer, which was later diagnosed as non-Hodgkin’s lymphoma (NHL) on biopsy and managed with chemotherapy successfully.

## 2. Case History

**Figure 1.**
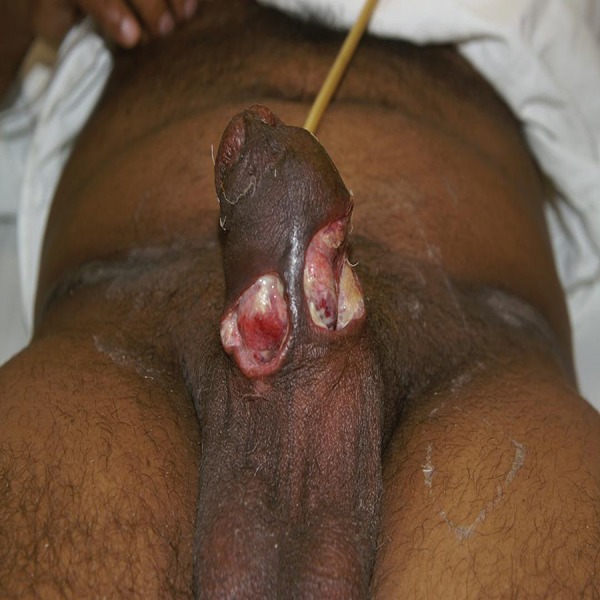
The Location and Size of Two Non-healing Ulcers Overlying the Nodule at the Base of Penis, Which Developed Following Incision and Drainage of the Lesion

A 49-year-old sexually active male presented to us with the complaint of swelling on the ventral surface of the penis near the base, double stream and intermittent flow of urine with post-micturition dribbling. He denied any history of sexually transmitted infection, discharge per urethra or trauma to the penis. His hematological, urinary and renal function parameters were normal. Retrograde urethrogram showed a small stricture in the posterior part of the penile urethra. Urethroscopy was planned but was postponed due to the development of pyuria and suprapubic cystostomy was done. He was last to follow-up and underwent urethral dilation at some other hospital. He presented again to us nine months later with an increase in the size of the swelling which was tender and fluctuant at this time. Incision and drainage were done. He, however, developed a non-healing ulcer at the site of swelling with leakage of urine from the shaft wound and dribbling of urine per urethra. Physical examination revealed 3 × 2 cm nodule just above the base of the penis which developed into two non-healing ulcers (Figure 1). The two ulcers measured 1 × 1.5 cm and 2 × 2 cm and were located on the ventral surface of the penis overlying the firm mass. The ulcers’ bases were firm, non-tender and exposed the underlying corpus spongiosum. There was diffuse swelling of the penis. There was no inguinal lymphadenopathy. Rest of the physical examination was unremarkable. The hematological and biochemical parameters were within normal limits. Magnetic resonance imaging (MRI) of the penis showed diffuse inflammatory involvement of the prostatic, bulbous, and penile urethra with predominance in the penile part.

**Figure 2.**
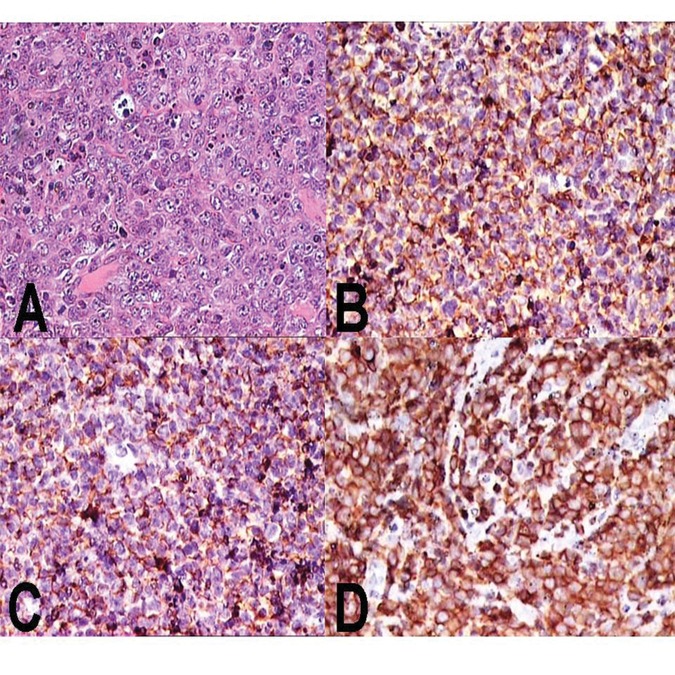
Biopsy Findings in the Case (A). Light microscopy showing diffuse sheets of large lymphoid cells with abundant cytoplasm, large vesicular nuclei with prominent nucleoli and scattered mitoses and apoptotic bodies (H&E, ×400) (B). Diffuse positivity of tumor cells for leukocyte common antigen (LCA) (IHC, ×400). (C). Diffuse positivity of tumor cells for CD20 (IHC, ×400). (D). Diffuse positivity of tumor cells for CD79a (IHC, ×400).

Penile corporal biopsy was performed in view of the non-healing ulcer and showed diffuse large B cell non-Hodgkin’s lymphoma (DLBCL). The tumor showed diffuse positivity for LCA (leukocyte common antigen), CD20 and CD7a in large lymphoid cells, while T cell markers showed positivity in sprinkled mature T lymphocytes in the background (Figure 2 A-D). The tumor cells were negative for CD5, CD10, bcl 2, Cyclin D1, CD15, and CD30. Ki-67 labeling revealed positivity in 60% of the tumor cells. The tumor cells were negative for epithelial, mesenchymal, and melanoma markers.

The complete hematological evaluation of the patient showed; hemoglobin 12.3 g/dL, red cell count 5.1 × 1012/L, packed cell volume 39.4%, MCV 77.9 fl, MCH 24.3 pg, MCHC 31.2 g/dL, total leucocyte count 12.5 × 109/L, neutrophils 88.4%, lymphocytes 5.8%, monocytes 5.4%, eosinophils 0.2% and basophils 0.2%. Platelets were 393 × 109/L. No blast cells were seen on peripheral blood smear examination. Serum LDH was 165 U/L (normal: 100-190 U/L).

Computerized tomography (CT) scan of the abdomen, pelvis and chest, and bone marrow biopsy were done for staging work-up. All these tests were negative for tumor involvement. Echocardiography was done as pretreatment assessment for chemotherapy tolerance and was normal. Patient was managed with CHOP (Cyclophosphamide, Doxyrubicin, Oncovir, Prednisolone) chemotherapy. The size of the ulcer and the diffuse swelling of the penis decreased significantly after the first cycle of chemotherapy. He has completed the six cycles with complete resolution of the disease with no significant side effects.

## 5. Discussion

The diagnosis of the rare diseases has always been a challenging process. There are many diagnostic dilemmas during this process. Lymphoma of the penis can present as a nodule, non-healing ulcer and diffuse penile swelling but no pathognomonic signs or symptoms have been reported (4). Lymphoma in this location is an indolent disease. Our patient presented with all the above three presenting features and was diagnosed accurately approximately one year after initial presentation and the disease was still localized. Penile shaft is the most common site of presentation of lymphoma followed by the glans penis (5). In the present case, after an early consideration of the differential diagnoses of Peyronie`s disease, and periurethral abscess, the final diagnosis of NHL of the penis was made on the histopathological examination of tissue biopsy. A representative and adequate biopsy of any non-healing ulcer is of utmost importance. Ultrasound-guided biopsy can also diagnose the disease (6). Histological analysis must include immunohistochemical tests to differentiate lymphoma from undifferentiated sarcomas or carcinomas and to distinguish between B- and T-cell lymphomas (7). Preservation of sexual function should always be addressed during the management of the patient. In this case, after the chemotherapy, the disease has been completely controlled. Chemotherapy is the preferred modality of treatment because of good cosmetic and functional outcome (8-10). Surgical option including radical surgery should be used only after the failure of other modalities (7).

In conclusion, lymphoma should be considered in any nodule or non-healing ulcer in the shaft of the penis.
